# Hepatitis B Surface Antigen Screening Among Pregnant Women and Care of Infants of Hepatitis B Surface Antigen–Positive Mothers — Guam, 2014

**DOI:** 10.15585/mmwr.mm6619a5

**Published:** 2017-05-19

**Authors:** Winston E. Abara, Susan Cha, Tasneem Malik, Mia S. DeSimone, Bernadette Schumann, Esther Mallada, Michael Klemme, Vince Aguon, Anne Marie Santos, Melissa Collier, Mary Kamb

**Affiliations:** ^1^Epidemic Intelligence Service, CDC; ^2^Division of Viral Hepatitis, CDC; ^3^Division of Sexually Transmitted Diseases Prevention, CDC; ^4^Division of Scientific Education and Professional Development, CDC; ^5^Emory University School of Medicine, Atlanta, Georgia; ^6^Guam Department of Public Health and Social Services; ^7^Guam Memorial Hospital Authority.

Hepatitis B virus (HBV) infection is endemic among adults in the U.S. territory of Guam (*1*,*2*). Perinatal HBV transmission, which occurs at birth from an infected mother to her newborn infant, is a major mode of HBV transmission and maintains HBV endemicity (*3*). Approximately 90% of HBV-infected infants will develop chronic HBV infection, and approximately 25% of those will die prematurely from liver failure or hepatocellular carcinoma (*4*,*5*). Since 1988, the Advisory Committee on Immunization Practices has recommended that all pregnant women be screened for hepatitis B surface antigen (HBsAg), an indicator of HBV infection, and that infants of women who screen positive (HBsAg-positive women) receive postexposure prophylaxis (PEP) (hepatitis B vaccine and hepatitis B immunoglobulin [HBIG]). When received within 12 hours of birth, PEP is 85%–95% effective in preventing perinatal HBV transmission (*5*,*6*). Hepatitis B vaccine provides long-term active immunity to HBV infection and HBIG provides short-term passive immunity to HBV infection until the infant responds to the vaccine (*5*). Hepatitis B vaccine was introduced into the routine universal infant vaccination schedule in Guam in 1988 (*1*).

Data for this analysis were obtained from the medical records of pregnant women who delivered live-born infants at Guam Memorial Hospital in 2014. This hospital is the largest delivery hospital in Guam and accounted for approximately 73% of all recorded births in 2014. Among 2,478 live-born infants delivered at this hospital during 2014, a sample of 971 (39%) was randomly selected. After excluding one infant from each of the five sets of twins in the selected sample, the final analytical sample consisted of 966 mother-infant pairs. Prenatal medical records of mothers of all 966 infants and vaccination records of infants of HBsAg-positive women were reviewed. Maternal demographic and clinical care data as well as information on the administration of hepatitis B vaccine and HBIG to infants of HBsAg-positive women were collected using a standardized chart abstraction tool. Descriptive analyses and frequencies were performed to calculate the prevalence of prenatal HBsAg screening, HBsAg positivity, demographic characteristics, prenatal care among pregnant women and the administration of hepatitis B vaccination and HBIG to infants of HBsAg-positive women. Receipt of prenatal care was defined as having ≥1 prenatal care visit before admission for delivery, and prenatal HBsAg screening was defined as documentation of testing for HBsAg at any time before birth, including during the delivery admission.

Among the 966 women in this sample, 752 (78%) were Pacific Islanders, 197 (21%) were Asian, 11 (1%) were white, and two (<1%) were Hispanic (Table). The mean and median age at delivery was 27 years (range = 15–45 years); 542 (56.1%) women were aged >25 years at delivery. Information on prenatal HBsAg screening was available for 936 (97%) women, 905 (97%) of whom received prenatal HBsAg screening. Overall, 857 (89%) women received prenatal care; among this group, prenatal HBsAg screening information was available for 834 (97%) women, 818 (98%) of whom were screened for HBsAg. Among the 106 (11%) women who did not receive prenatal care, prenatal HBsAg screening data were available for 102 (96%); among these women, 87 (85%) were screened for HBsAg upon admission for delivery. The odds of receiving HBsAg screening among women who received prenatal care was significantly higher than among those who did not receive prenatal care (odds ratio = 8.82, p<0.001).

**TABLE:** Demographic characteristics, prenatal hepatitis B surface antigen (HBsAg) screening, prenatal care received, and screening results among a random sample of pregnant women with live-born deliveries, and receipt of hepatitis B virus postexposure prophylaxis among infants of HBsAg-positive mothers — Guam Memorial Hospital, Guam, 2014 (N = 966)

Characteristic	No. (%)*
**Race/Ethnicity (N = 962)**	
Pacific Islander	752 (78.2)
Asian	197 (20.5)
White	11 (1.1)
Hispanic	2 (0.2)
**Prenatal HBsAg screening received^†^ (N = 936)**	
Yes	905 (96.7)
No	31 (3.3)
**Prenatal care received^§^ (N = 963)**	
Yes	857 (89.0)
No	106 (11.0)
**Prenatal HBsAg screening among women with prenatal care (N = 834)**	
Yes	818 (98.1)
No	16 (1.9)
**Prenatal HBsAg screening among women without prenatal care (N = 102)**	
Yes	87 (85.3)
No	15 (14.7)
**Maternal HBsAg screening results (N = 899)**	
HBsAg-positive	18 (2.0)
HBsAg-negative	881 (98.0)
**Receipt of postexposure HBV prophylaxis among infants born to HBsAg-positive women^¶^ (N = 18)**	
Received HB vaccine within 12 hrs of delivery	18 (100)
Received HBIG within 12 hrs of delivery	17 (94)
**Age at delivery, yrs (N = 966)**	
Mean	27.2
Median	27.0
Range (SD)	15–45 (6.2)
>25 yrs (all mothers [N = 966])	542 (56.1)
>25 yrs (HBsAg-positive mothers [N = 18])	16 (88.9)

**Abbreviations:** HBIG = hepatitis B immune globulin; HBV = hepatitis B virus; SD = standard deviation.

* Except as noted.

**^†^** Includes women screened during prenatal care and women without prenatal care who were screened upon admission for delivery.

**^§^** At least one prenatal care visit before delivery.

**^¶^** Limited to infants born to HBsAg-positive mothers.

Among 899 women with available HBsAg screening result data, 18 (2%) were HBsAg-positive, of whom 14 were Pacific Islanders and four were Asian. Sixteen (89%) HBsAg-positive women were aged >25 years of age at delivery (born before the introduction of hepatitis B vaccine into the routine immunization program in 1988), and were therefore less likely to have been vaccinated against hepatitis B as infants; hepatitis B vaccination status of mothers was not available. All 18 infants born to HBsAg-positive women received hepatitis B vaccination within 12 hours of delivery and 17 of 18 received HBIG.

## Discussion

The prevalence of prenatal HBsAg screening in this hospital-based random sample of women with a live birth during 2014 in Guam (97%) was similar to the 94% prevalence estimate in the continental United States in 2010 (*7*); however, the 2.0% HBsAg positivity prevalence in this sample is approximately 13 times higher than the 0.14% maternal prevalence estimate among U.S.-born Pacific Islander and Asian women and approximately twice the 0.9% maternal prevalence estimate in the continental United States (*7*,*8*). Despite the high HBsAg prevalence in this sample, all infants born to HBsAg-positive women received hepatitis B vaccine, and all but one infant received HBIG. Most of the HBsAg-positive women included in the sample were born before hepatitis B vaccine was introduced into Guam’s universal infant vaccination schedule; although catch-up vaccination programs were implemented in later years, many women might have been missed. However, infant and childhood hepatitis B vaccination coverage has significantly increased in Guam and the other U.S-affiliated Pacific Islands since the introduction of universal infant hepatitis B vaccination (*9*). The estimated hepatitis B vaccine coverage among children in this region is 98%; consequently, the risk for perinatal HBV transmission is likely to decrease in future birth cohorts (*9*).

The findings in this report are subject to at least two limitations. First, although this hospital accounted for approximately three quarters of births registered in 2014 in Guam, the data came from only one hospital; therefore, the prevalence of prenatal HBsAg screening, maternal HBsAg positivity, hepatitis B vaccination, and HBIG administration cannot be generalized to all health care facilities in Guam. Second, no postvaccination serologic testing data for the 18 infants born to HBsAg-positive mothers were available to assess HBV infection and immune response status, although administration of hepatitis B vaccine and HBIG within 12 hours of birth is reported to be 85%–95% effective in preventing perinatal transmission (*5*).

The prevalence of HBsAg screening among women who had at least one prenatal care visit (98%) was significantly higher than that among women who did not (85%). Prenatal screening of all pregnant women for HBsAg is a critical component of the HBV elimination strategy in the United States and its territories (*6*), especially in areas with a high prevalence of HBV infection in adults. Prenatal screening facilitates the timely identification of HBsAg-positive women and ensures that PEP is available to their infants immediately after delivery, thus reducing the likelihood of infants becoming chronically infected and serving as reservoirs for continued HBV transmission (*5*,*10*). Prenatal care is important for prenatal HBSAg screening in Guam. Fully implementing systemic and institutional hospital policies that require documentation of maternal HBsAg status in hospital maternity records and the administration of PEP to all infants of HBsAg-positive mothers will ensure that all infants at risk receive PEP and that the risk for perinatal HBV transmission is reduced (*7*).

**Summary**

**What is already known about this topic?**

Hepatitis B virus (HBV) infection is endemic in the U.S. territory of Guam, and perinatal transmission is a major mode of transmission. The Advisory Committee on Immunization Practices recommends that all pregnant women be screened for hepatitis B surface antigen (HBsAg) in each pregnancy and that infants of HBsAg-positive women receive postexposure prophylaxis (PEP) with hepatitis B vaccine and hepatitis B immune globulin (HBIG) within 12 hours of birth to reduce the risk for perinatal HBV transmission.

**What is added by this report?**

In a hospital-based random sample of women with a live birth during 2014 in Guam, HBsAg seroprevalence (2.0%) was approximately 13 times higher than that among U.S.-born Pacific Islander and Asian women (0.14%) and approximately twice the overall U.S. maternal prevalence estimate (0.9%). Approximately 90% of HBsAg-positive women were born before introduction of universal infant hepatitis B vaccination. Among women who had at least one prenatal care visit, 98% received prenatal HBsAg screening, compared with 85% of women who did not receive prenatal care. All infants of HBsAg-positive women received hepatitis B vaccine and all but one infant received HBIG.

**What are the implications for public health practice?**

Prenatal HBSAg screening facilitates prompt identification of HBsAg-positive pregnant women and mitigates the risk for perinatal HBV transmission. Timely administration of PEP to infants of HBsAg-positive women is important to prevent perinatal HBV transmission.
